# Inhibitory effect of 4-*O*-methylhonokiol on lipopolysaccharide-induced neuroinflammation, amyloidogenesis and memory impairment via inhibition of nuclear factor-kappaB *in vitro *and *in vivo *models

**DOI:** 10.1186/1742-2094-9-35

**Published:** 2012-02-19

**Authors:** Young-Jung Lee, Dong-Young Choi, Im Seop Choi, Ki Ho Kim, Young Hee Kim, Hwan Mook Kim, Kiho Lee, Won Gil Cho, Jea Kyung Jung, Sang Bae Han, Jin-Yi Han, Sang-Yoon Nam, Young Won Yun, Jae Hwang Jeong, Ki-Wan Oh, Jin Tae Hong

**Affiliations:** 1 College of Pharmacy, Chungbuk National University, 12, Gaeshin-dong, Heungduk-gu, Cheongju, Chungbuk 361-763, Korea; 2 Medical Research Center, Chungbuk National University, 12 Gaesin-dong, Heungduk-gu, Cheongju, Chungbuk 361-763, Korea; 3 R&D Center, Bioland Ltd., Songjeong, Byongchon, Cheonan-Si, Chungnam 330-863, Korea; 4 College of Pharmacy, Gachon University of Medicine and Science, Incheon 406-799, Korea; 5 Wonju College of Medicine, Yonsei University, 162 Ilsan-dong, Wonju-si, Ganwon-do 220-701, Korea; 6 College of Veterinary Medicine and Research Institute of Veterinary Medicine (RIVM), Chungbuk National University, 12 Gaesin-dong, Heungduk-gu, Cheongju, Chungbuk 361-763, Korea; 7 Department of Biotechnology and Bioinformatics, Chungbuk Provincial College of Science & Technology, Okcheon, Chungbuk 373-807, Korea

**Keywords:** Alzheimer's disease, Amyloid, Lipopolysaccharide, Neuroinflammation, 4-*O*-methylhonokiol, *Magnolia officinalis*, Memory impairment

## Abstract

**Background:**

Neuroinflammation is important in the pathogenesis and progression of Alzheimer disease (AD). Previously, we demonstrated that lipopolysaccharide (LPS)-induced neuroinflammation caused memory impairments. In the present study, we investigated the possible preventive effects of 4-*O*-methylhonokiol, a constituent of *Magnolia officinalis*, on memory deficiency caused by LPS, along with the underlying mechanisms.

**Methods:**

We investigated whether 4-*O*-methylhonokiol (0.5 and 1 mg/kg in 0.05% ethanol) prevents memory dysfunction and amyloidogenesis on AD model mice by intraperitoneal LPS (250 μg/kg daily 7 times) injection. In addition, LPS-treated cultured astrocytes and microglial BV-2 cells were investigated for anti-neuroinflammatory and anti-amyloidogenic effect of 4-*O*-methylhonkiol (0.5, 1 and 2 μM).

**Results:**

Oral administration of 4-*O*-methylhonokiol ameliorated LPS-induced memory impairment in a dose-dependent manner. In addition, 4-*O*-methylhonokiol prevented the LPS-induced expression of inflammatory proteins; inducible nitric oxide synthase (iNOS) and cyclooxygenase-2 (COX-2) as well as activation of astrocytes (expression of glial fibrillary acidic protein; GFAP) in the brain. In *in vitro *study, we also found that 4-*O*-methylhonokiol suppressed the expression of iNOS and COX-2 as well as the production of reactive oxygen species, nitric oxide, prostaglandin E_2_, tumor necrosis factor-α, and interleukin-1β in the LPS-stimulated cultured astrocytes. 4-*O*-methylhonokiol also inhibited transcriptional and DNA binding activity of NF-κB via inhibition of IκB degradation as well as p50 and p65 translocation into nucleus of the brain and cultured astrocytes. Consistent with the inhibitory effect on neuroinflammation, 4-*O*-methylhonokiol inhibited LPS-induced Aβ_1-42 _generation, β- and γ-secretase activities, and expression of amyloid precursor protein (APP), BACE1 and C99 as well as activation of astrocytes and neuronal cell death in the brain, in cultured astrocytes and in microglial BV-2 cells.

**Conclusion:**

These results suggest that 4-*O*-methylhonokiol inhibits LPS-induced amyloidogenesis via anti-inflammatory mechanisms. Thus, 4-*O*-methylhonokiol can be a useful agent against neuroinflammation-associated development or the progression of AD.

## Background

Alzheimer's disease (AD) is the most common cause of dementia accounting for 50% to 75% of all cases [1,2]. AD is pathologically characterized by the presence of senile plaques and neurofibrillary tangles in the brain. In particular, the senile plaques are extracellular aggregates of the amyloid beta-peptide (Aβ) that is cleaved from the amyloid precursor protein (APP) [3]. Neuropathological studies in the human brains have demonstrated that the activation of glial cells excessively releases pro-inflammatory mediators and cytokines, which in turn trigger a neurodegenerative cascades via neuroinflammation [4-8]. Numerous investigators have reported that neuroinflammatory processes contribute to the pathogenesis and progression of AD. In neuroinflammation, various cytokines, chemokines, oxygen free radicals, and reactive nitrogen species [9], as well as prostaglandin E_2 _(PGE_2_) [10], are important signaling molecules and components of neuroinflammatory responses [11].

It has also been shown that the inflammatory mediators such as Nitric oxide (NO) and prostaglandins (PGs) as well as cytokines such as interleukin (IL)-1β, IL-6, tumor necrosis factor-α (TNF-α) and transforming growth factor-β (TGF-β) can augment APP expression [12,13] and Aβ formation [14]. These inflammatory mediators and cytokines are able to transcriptionally upregulate β-secretase mRNA, protein, and enzymatic activity [15], thus affecting Aβ formation [16]. However, anti-inflammatory compounds decrease memory deficiency and the accumulation of Aβ plaques, and elevate levels of soluble APP-α [17,18]. Moreover, the administration of anti-inflammatory agents in AD patients could reduce amyloidogenesis, suggesting that neuroinflammation may influence the occurrence and pathogenesis of AD through anti-amyloidogenesis [19].

Systemic administration of lipopolysaccharides (LPS) induces cognitive impairment in mice [20,21]. The administration of LPS also induces the release of the proinflammatory cytokines such as IL-1β, IL-6, and TNF-α and these cytokines exert neurobiological effects [22], suggesting that induction of systemic inflammation affects the neurobiological condition. There is much evidence illustrating that LPS-induced neuroinflammation is related to the up-regulation of NF-κB [23-26]. LPS-induced neuronal damages can be restored via the inhibition of IκB kinase-β [23]. In our previous study, we showed that intraperitoneal (i.p.) injections of LPS induced memory impairment and amyloidogenesis in *in vivo*, and anti-inflammatory compounds such as (-)-epigallocatechin-3-gallate (EGCG) abolished LPS-induced amyloidogenesis via inhibiting NF-κB as well as inhibiting β- and γ-secretase activities [27,28]. Thus, systemic administration of LPS could be applicable for the study of neuroinflammation and neuroinflammation-associated pathogenesis of AD.

*Magnolia *bark has been used in traditional medicine to treat various disorders [29,30]. Several constituents of the *Magnolia *such as honokiol, obovatol and magnolol have been reported to have anti-inflammatory [31-33], neuroprotective [34-36], and anti-oxidative effects [37,38]. Recently, we found that 4-*O*-methylhonokiol isolated from *Magnolia officinalis *has anti-oxidative [39], anti-inflammatory [40] and neurotrophic activities [41]. It also showed memory-improving effects via the reduction of Aβ accumulation in Aβ_1-42_-injected mice and memory-deficient transgenic mice via the anti-oxidative and anti-inflammatory effects [42-44]. In this study, to define the effect of 4-*O*-methylhonokiol against Aβ accumulation via the prevention of neuroinflammation, we investigated the effect of 4-*O*-methylhonokiol on LPS-induced memory impairments and amyloidogenesis via anti-inflammatory reactions in LPS-injected mice brain, in cultured astrocytes and in microglial BV-2 cells.

## Methods

### 4-*O*-methylhonokiol

**Figure 1:**
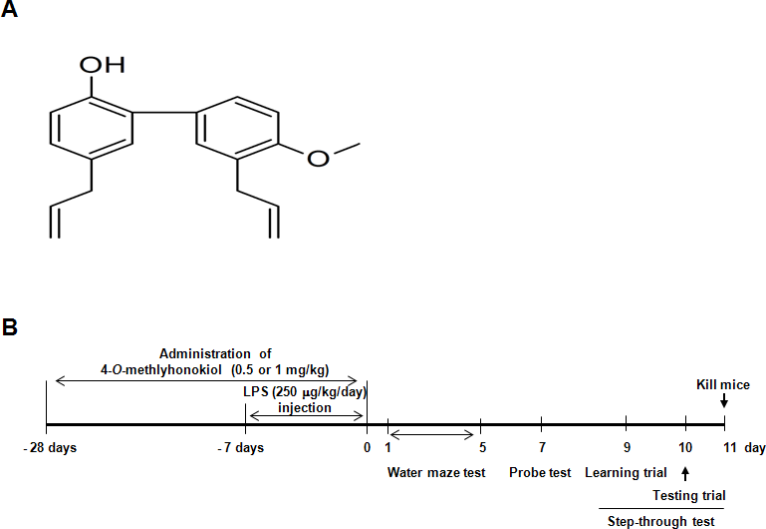
**Chemical structure of 4-*O*-methylhonokiol (A) and experimental scheme (B)**.

2-[4-Methoxy-3-(2-propenyl)phenyl]-4-(2-propenyl)phenol (4-*O*-methylhonokiol, Molecular Weight = 280.4, Molecular Formula = C_19_H_20_O_2_) of chemical structure shown in Figure 1A was isolated from the bark of *Magnolia officinalis *as described elsewhere [40,45]. The bark of *Magnolia officinalis *was dried in the shade at room temperature and stored in a dark, cold room until use. The air-dried bark of *Magnolia officinalis *(3 kg) was cut into pieces and extracted twice with 95% (v/v) ethanol (four times as much as the weight of the dried plants) for 3 days at room temperature. After filtration through the 400-mesh filter cloth, the filtrate was re-filtered through filter paper (Whatman, No. 5) and concentrated under reduced pressure. The extract (450 g) was then suspended in distilled water, and the aqueous suspension was extracted with n-hexane, ethyl acetate, and n-butanol, respectively. The n-hexane layer was evaporated to dry, and the residue (70 g) was chromatographed on silica gel with n-hexane:ethyl acetate (9:1) solution to extract a crude fraction that included 4-*O*-methylhonokiol. This fraction was repeatedly purified by silica gel chromatography using n-hexane:ethyl acetate as the eluent to obtain pure 4-*O*-methylhonokiol (Figure 1A). The purity was more than 99.5%. 4-*O*-methylhonokiol was identified by 1H NMR (400 MHz, CDCl3) I: 3.36 (2H, d, J = 7 Hz, H-7), 3.44 (2H, d, J = 7 Hz, 7'-H), 3.89 (3H, s, OMe), 5.05-5.14 (5H, m, H-9, H-9', OH), 5.93-6.07 (2H, m, H-8, H-8'), 6.92 (1H, d, J = 7 Hz, Ar-H), 6.97 (1H, d, J = 8 Hz, Ar-H), 7.04-7.08 (2H, m, Ar-H), 7.24-7.31 (2H, m, Ar-H) and 13 C NMR (100 MHz, CDCl3) I: 34.5 (C-7), 39.6 (C-7'), 55.8 (OMe), 111.2 (C-3'), 115.7 (C-4'), 115.8 (C-9), 116.1 (C-9'), 128.0 (C-1'), 128.1 (C-6), 129.0 (C-3), 129.2 (C-1), 130.0 (C-5), 130.4 (C-6'), 130.7 (C-2), 132.4 (C-5'), 136.7 (C-8), 138.0 (C-8'), 151.0 (C-2'), 157.2 (C-4). The ethanol extract of *Magnolia officinalis *contained 16.6% 4-*O*-methylhonokiol followed by 16.5% honokiol and 12.9% magnolol, and 42-45% others. The information of 4-*O*-methylhonokiol was previously reported [40,45]. The result was in agreement with previously published data [46,47], and this compound may possibly be the same compound demonstrated by Zhou et al. [35].

Dosage (0.5 and 1 mg/kg/day) of 4-*O*-methylhonokiol in this study was used by referring to our previous studies [43-45,48]. 4-*O*-methylhonokiol (15 and 30 μg/mouse) was added to drinking water (5 ml of average water consumption of mouse per day) and mice were allowed access for 3 weeks *ad libitum *before induction of memory impairment as shown in Figure 1B.

### Lipopolysaccharide-induced memory impairment mouse model

Five week-old male imprinting control region (ICR) mice (Samtako, Gyeonggi-do, Korea) were maintained in accordance with the Institutional Animal Care and Use Committee (IACUC) of Laboratory Animal Research Center at Chungbuk National University, Korea (CBNUA-144-1001-01). All mice were housed in a room that was automatically maintained at 21-25°C and relative humidity (45-65%) with a controlled light-dark cycle. Several researchers reported that repeated i.p. injection of LPS induced cognitive impairment like AD in mice [21,49-51]. We therefore used this method as an AD mice model. The LPS (Sigma, St. Louis, MO. USA, final concentration of 0.1 mg/ml) was dissolved, and aliquots in saline were stored at -20°C until use. The i.p. injection (250 μg/kg) of LPS or control (saline) was daily administered for 7 days. Subsequently, the behavioral tests of learning and memory capacity were assessed using two separate tests (water maze and passive avoidance test). One day interval was given between tests for adaptation of new circumstances as shown in Figure 1B.

### Water maze test

The water maze test is also a widely accepted method for memory test, and we performed this test as described by Morris et al. [52]. Maze testing was performed by the SMART-CS (Panlab, Barcelona, Spain) program and equipment. A circular plastic pool (height: 35 cm, diameter: 100 cm) was filled with milky water kept at 22-25°C. An escape platform (height: 14.5 cm, diameter: 4.5 cm) was submerged 0.5-1 cm below the surface of the water in position. On training trials, the mice were placed in a pool of water and allowed to remain on the platform for 10 s and were then returned to the home cage during the second-trial interval. The mice that did not find the platform within 60 s were placed on the platform for 10 s at the end of trial. They were allowed to swim until they sought the escape platform. These trials were performed in single platform and in three starting positions of rotational starting. Escape latency, escape distance, swimming speed and swimming pattern of each mouse was monitored by a camera above the center of the pool connected to a SMART-LD program (Panlab, Barcelona, Spain).

### Probe test

A probe trial in order to assess memory consolidation was performed 24 h after the 5-day acquisition tests. In this trial, the platform was removed from the tank, and the mice were allowed to swim freely. For these tests, the percentage time in the target quadrant and target site crossings within 60 s was recorded. The time spent in the target quadrant is taken to indicate the degree of memory consolidation that has taken place after learning. The time spent in the target quadrant was used as a measure of spatial memory. Swimming pattern of each mouse was monitored by a camera above the center of the pool connected to a SMART-LD program described above.

### Passive avoidance performance test

The passive avoidance test is widely accepted as a simple and rapid method for memory test. The passive avoidance response was determined using a "step-through" apparatus (Med Associates Inc, Vermont, USA) that is consisted of an illuminated and a dark compartment (each 20.3 × 15.9 × 21.3 cm) adjoining each other through a small gate with a grid floor, 3.175 mm stainless steel rod set 8 mm apart. Two days after the water maze test, the ICR mice were placed in the illuminated compartment facing away from the dark compartment for the training trial. When the mice moved completely into the dark compartment, it received an electric shock (2 mA, 3 s duration). Then the mice were returned to their home case. 24 h later, the mice were placed in the illuminated compartment and the latency period to enter the dark compartment defined as "retention" was measured. The time when the mice entered in the dark compartment was recorded and described as step-through latency. The retention trials were set at a limit of 180 s of cutoff time.

### Astrocytes and microglial BV-2 cells culture

As described elsewhere [53,54], 2-day-olds rat pups were ice-anesthetized and decapitated. After the skull was cut and the skin was opened, the brain was released from the skull cavity. After washing with PBS, the cerebrum was separated from the cerebellum and brain stem, and the cerebral hemispheres were separated from each other by gently teasing along the midline fissure with the sharp edge of forceps. The meninges were gently peeled from the individual cortical lobes and the cortices were dissociated by mechanical digestion [using the cell strainer (BD Bioscience, Franklin Lakes, NJ, USA)] with Dulbecco's modified Eagle's medium (DMEM) containing F12 nutrient mixture (Invitrogen, Carlsbad, CA). The resulting cells were centrifuged (1,500 rpm, 5 min), resuspended in serum-supplemented culture media, and plated into 100 mm dishes. Serum-supplemented culture media was composed of DMEM supplemented with F12, FBS (5%), NaHCO_3 _(40 mM), penicillin (100 units/ml), and streptomycin (100 μg/ml). The cells were incubated in the culture medium in a humidified incubator at 37°C and 5% CO_2 _for 9 days. At confluence (9 days), the flask was subjected to shaking for 16-18 h at 37°C. The cultures were treated for 48 h with cytosine arabinoside and the medium was replaced with DMEM/F12HAM containing 10% FBS. The monolayer was treated with 1.25% trypsin-EDTA for a short duration after which the cells were dissociated and plated into uncoated glass coverslips. The astrocyte cultures formed a layer of process-bearing, glial fibrillary acidic protein (GFAP)-positive cells. The purity of astrocyte cultures was assessed by GFAP-immunostaining. Under these conditions, we can assume that over 95% of the cells were astrocytes. Microglial BV-2 cells were maintained with serum-supplemented culture media of DMEM supplemented with FBS (5%), NaHCO_3 _(40 mM), penicillin (100 units/ml), and streptomycin (100 μg/ml). The BV-2 cells were incubated in the culture medium in a humidified incubator at 37°C and 5% CO_2_. The cultured cells were treated simultaneously with LPS (1 μg/ml) and several concentrations (0.5, 1, 2 μM) of 4-*O*-methylhonokiol dissolved in 0.05% ethanol, and the cells were harvested after 24 h. Western blotting was performed, and Aβ level and secretases activities were determined.

### Nitric oxide and PGE_2 _determination

Astrocytes were grown in 96-well plates and then incubated with or without LPS (1 μg/ml) in the absence or presence of various concentrations of 4-*O*-methylhonokiol for 24 h. The nitrite accumulation in the supernatant was assessed by Griess reaction [55]. Each 50 μl of culture supernatant was mixed with an equal volume of Griess reagent [0.1% N-(1-naphthyl)-ethylenediamine, 1% sulfanilamide in 5% phosphoric acid] and incubated at room temperature for 10 min. The absorbance at 540 nm was measured in a microplate absorbance reader, and a series of known concentrations of sodium nitrite was used as a standard. In the cultured supernatant of astrocytes, PGE_2 _concentration was determined using a PGE_2 _Enzyme Immunometric Assay (EIA) kit (R&D systems), according to the manufacturer's instructions.

### Reactive oxygen species (ROS) generation

To monitor intracellular accumulation of ROS in cultured astrocytes, the fluorescent probe 2',7'-dichlorofluorescein diacetate (DCF-DA) was used. Following treatment with LPS (1 μg/ml) for 24 h in the presence or absence of 4-*O*-methylhonokiol (0.5, 1, 2 μM), the cells were washed in modified Kreb's buffer containing 145 mM NaCl, 5 mM potassium chloride (KCl), 1 mM magnesium chloride (MgCl_2_), 1 mM calcium chloride (CaCl_2_), 4 mM sodium hydrogen carbonate (NaHCO_3_), 5.5 mM glucose, 10 mM HEPES, pH 7.4. The cell suspension was transferred into plastic tubes. Measurement was started by an injection of 5 μM DCF-DA in the dark. After 30 min of incubation at 37°C, generation was determined by Fluorometer (fmax, Molecular devices corp., Sandiego, CA, USA) at Ex = 485 and Em = 538 nm.

### Measurement of Aβ_1-42 _and Aβ_1-40 _level

Lysates of brain tissue, astrocytes and microglial BV-2 cells were obtained through protein extraction buffer containing protease inhibitor. Aβ_1-42 _levels were determined using a specific ELISA Kit (Immuno-Biological Laboratories Co., Ltd., Takasaki-Shi, Gunma, Japan). In brief, 100 μl of sample was added into the pre-coated plate and was incubated overnight at 4°C. After washing each well of the precoated plate with washing buffer, 100 μl of labeled antibody solution was added and the mixture was incubated for 1 h at 4°C in the dark. After washing, chromogen was added and the mixture was incubated for 30 min at room temperature in the dark. Finally, the resulting color was assayed at 450 nm using a microplate absorbance reader (SunriseTM, TECAN, Switzerland) after adding stop solution.

### β- and γ-secretase assay

The total activities of β- and γ-secretase in the brains and astrocytes were measured using a commercially available β-secretase (BACE1) fluorescence resonance energy transfer assay kit (PANVERA, Madison, USA) and γ-secretase activity kit (R&D Systems, Wiesbaden, Germany) according to the manufacturers' protocols and as described elsewhere [28]. The brains and astrocytes were homogenized in cold 1× cell extraction buffer (ready for use in the kit) to yield a final protein concentration of 1 mg/ml.

To determine β-secretase activity, 10 μl of lysate was mixed with 10 μl of BACE1 substrate (Rh-EVNLDAEFK-Quencher), and then the reaction mixture was incubated for 1 h at room temperature in the 96 well flat bottom microtitre plate. The reaction was stopped by the addition of 10 μl of BACE1 stop buffer (2.5 M sodium acetate). The formation of fluorescence was read with Fluostar galaxy fluorometer (excitation at 545 nm and emission at 590 nm) with Felix software (BMG Labtechnologies, Offenburg, Germany). The enzyme activity was linearly related to the increase in fluorescence. The enzyme activity was expressed as nM produced substrate which was determined by the formation of fluorescence per mg protein per min. All controls, blanks and samples were run in triplicate.

To determine γ-secretase activity, 50 μl of lysate was mixed with 50 μl of reaction buffer. The reaction mixture was then incubated for 1 h in the dark at 37°C followed by the addition of 5 μl of substrate. Cleaved substrate by γ-secretase was conjugated to the reporter molecules EDANS and DABCYL, and released fluorescent signal. This formation of fluorescence was read with Fluostar galaxy fluorometer (excitation at 355 nm and emission at 510 nm) with Felix software (BMG Labtechnologies, Offenburg, Germany). The level of γ-secretase enzymatic activity is proportional to the fluorometric reaction, and the γ-secretase activity was expressed as the produced fluoresce unit.

### Nuclear extraction and gel mobility shift assay

Gel mobility shift assay was conducted using a slight modification of a previously described method [44]. In brief, 10 μg of nuclear protein of astrocytes was incubated in 25 μL of total volume of incubation buffer (10 mmol/L Tris, pH 7.5, 100 mmol/L NaCl, 1 mmol/L dithiothreitol, 4% glycerol, 80 mg/L salmon sperm DNA) at 4°C for 15 min followed by another 20 min incubation with 9.25 mBq [γ-^32^P] ATP-labeled oligonucleotide containing the NF-κB binding site at room temperature. The DNA-protein binding complex was electrophoretically resolved on a 6% nondenatured polyacrylamide gel at 150 volts for 2 h. The gels were dried and autoradiographed using Kodak MR film at -80°C overnight.

### Quantitative real-time PCR

For mRNA quantification, total RNA was extracted using the RNAqueous kit (Applied Biosystems, Foster city, CA). The cDNA was synthesized using High Capacity RNA-to-cDNA kit (Applied Biosystems, Foster city, CA) according to the manufacturer's protocol. Briefly, 1 μg of total RNA was used for cDNA preparation. Quantitative real-time PCR was performed on cDNA using TaqMan Gene Expression Assays (Applied Biosystems, Foster City, CA) specific for glyceraldehyde-3-phosphate dehydrogenase (GAPDH; assay no. Mm99999915_g1), TNF-α (Mm00443258_m1) and IL-1β (Mm00434228_m1). All reverse transcription reactions were run in a 7500 Real-Time PCR System (Applied Biosystems, Foster city, CA) using the universal cycling parameters (20 s 95°C, 60 cycles of 3 s 95°C, 30 s 60°C). The values obtained for the target gene expression were normalized to GAPDH and quantified relative to the expression in control samples. For the calculation of relative quantification, the 2^-△△*C*T ^formula was used, where:

$$-\Delta \Delta \text{CT}=\left(C_{\text{T},\text{target}}-C_{\text{T},\text{GAPDH}}\right)\;\text{experimental}\;\text{sample}-\left(C_{\text{T},\text{target}}-C_{\text{T},\text{GAPDH}}\right)\;\text{control}\;\text{sample}.$$

### Western blotting

An equal amount of total protein (40 μg) was resolved on an SDS/10 or 15% polyacrylamide gel and then transferred to a polyvinylidene difluoride membrane (GE Water and Process Technologies, Trevose, PA, USA). The membrane was incubated at room temperature with specific antibodies. To detect target proteins, specific antibodies against BACE1 (1:500, Sigma, St. Louis, MO, USA), C99, APP (1:500, ABR-affinity Bioreagents, Golden, CO, USA), Aβ (1:500, 4G8, Covance, Berlely, CA, USA), cleaved caspase-3 (1:200, Cell Signaling Technology, Inc., Beverly, MA, USA), COX-2 (1:1000, Cayman Chemical, Ann Arbor, MI, USA), iNOS (1:1000, Abcam), p65, IκB, pIκB (1:500, Santa Cruz Biotechnology Inc. Santa Cruz, CA, USA) and p50 (1:500, Santa Cruz Biotechnology Inc. Santa Cruz, CA, USA) were used. The blot was then incubated with the corresponding conjugated anti-mouse IgG-horseradish peroxidase (1:4000, Santa Cruz Biotechnology Inc. Santa Cruz, CA, USA). Immunoreactive proteins were detected with an enhanced chemiluminescence western blotting detection system. The relative density of the protein bands was scanned by densitometry using MyImage (SLB, Seoul, Korea), and quantified by Labworks 4.0 software (UVP Inc., Upland, CA, USA).

### Immunohistochemistry and immunofluorescence

Brains were fixed in formalin and paraffin-enclosed for examination. Five-micrometer-thick tissue sections were used with immunohistochemistry. Paraffin-embedded sections were deparaffinized and rehydrated, washed in distilled water, and then subjected to heat-mediated antigen retrieval treatment. Endogenous peroxidase activity was quenched by incubation in 2% hydrogen peroxide in methanol for 15 min and then cleared in PBS for 5 min. The sections were blocked for 30 min with 3% normal horse serum diluted in PBS. These sections were incubated for overnight with appropriate antibodies; Aβ_1-42 _(1:2000, Clone No. 4 G8, Covance, Berkeley, CA, USA), GFAP (1:5000, Abcam, Inc, Cambridge, MA, USA), COX-2 (1:100, Cayman Chemical, Ann Arbor, MI, USA), iNOS (1:100, Abcam) and cleaved caspase-3 (1:200, Cell Signaling Technology, Inc.). After the incubation, sections were washed in PBS and incubated with the biotinylated secondary antibodies (ABC kit, Vector Laboratories, Burlingame, CA) for 1 h. The sections were washed with PBS, incubated with the avidin-biotin complex (Vector Laboratories) for 30 min, and visualized by chromogen DAB (Vector Laboratories) reaction. It was then counterstained by a hematoxylin. Finally, sections were dehydrated in ethanol, cleared in xylene, and mounted with Permount (Fisher Scientific, Hampton, NH), and evaluated on a light microscopy (Olympus, Tokyo, Japan). To determine the expression of iNOS, BACE, GFAP and cleaved caspase-3, the stained cells by each antibody were counted. The six sections with three different animal brains were analyzed, and cells at three randomly selected areas (100 × 100 μm) in each section were assessed. The immunoreactive cells by anti-iNOS, BACE, GFAP and cleaved caspase-3 antibody were counted, and expressed as percentage of stained cells. The quantity of reactive cells was expressed as the average number of reactive cells per high power field (visible reactive cells/HPF). To simultaneously determine level of GFAP and Aβ, we performed immunofluorescence assay in paraffin section of brain. The sections were then incubated to primary rabbit polyclonal antibody for GFAP (1:1000, Abcam, Inc, Cambridge, MA, USA) and mouse monoclonal antibody for Aβ_1-42 _(1:2000, Clone No. 4 G8, Covance, Berkeley, CA, USA) overnight at 4°C. After washes with ice-cold PBS, followed by treatment with an anti-rabbit secondary antibody labeled with Alexa Fluor 568 and anti-mouse secondary antibody labeled with Alexa Flour 488 (1:100 dilution, Molecular Probes, Inc., Eugene, OR) for 2 hr at room temperature, immunofluorescence images were acquired using a confocal laser scanning microscope (TCS SP2, Leica Microsystems AG, Wetzlar, Germany). Areas of amyloid deposition in mice brain were identified by staining of 0.2% congo-red (Sigma) solution or 0.2% thioflavine S (Sigma) as described in detail in Wilcock et al. [56] and microscopic evaluation. For quantification of congophilic plaque load, digital images were captured at 10 × magnification on an Olympus IX70 Imaging System using a single exposure setting as follows: the entire hippocampus (2 images); the visual (1 image), somatosensory (1 image) and somatomotor (1 image) cortex. Images were converted to gray scale and the threshold intensity was set to the intensity observed in areas without tissue. Plaque load was defined as the% area, i.e. the positive area/total area × 100. The data were expressed as the mean ± SEM (n = 6 for each group).

### Measurement of apoptotic cells

The terminal deoxynucleotidyltransferase (TdT)-mediated dUTP-biotin nick end-labeling (TUNEL) assays were performed by using the in situ cell death detection kit (Roche Diagnostics GmbH, Mannheim, Germany) according to the manufacturer's protocol. TUNEL mixture was added onto tissue sections and incubated in a humidified chamber for 60 min at 37°C. After each step, the tissue sections were rinsed twice with a phosphate-buffered saline (PBS, pH 7.4). For DAPI staining, brains were incubated for 30 min at room temperature in the dark. The cells were then observed through a fluorescence microscope (Leica Microsystems AG, Wetzlar, Germany). Total number of cells in given area was determined by using DAPI nuclear staining. The apoptotic bodies (TUNEL-stained cells) were identified under a fluorescencemicroscope (x200) containing green colored nuclei. The quantity of apoptotic bodies was expressed as the average number of apoptotic cells per high power field (visible apoptotic cells/HPF).

### Measurement of TNF-α and IL-1β

TNF-α and IL-1β concentrations were measured in supernatant of astrocytes using ELISAs specific for rat TNF-α, and IL-1β (Assay Designs, Ann Arbor, Michigan), respectively, according to the manufacturer's instructions. After samples and standards were added to wells, plates were incubated for 1 h at 37°C. Wells were washed 7 times with wash solution, at which point antibody was added to each well and incubated for 30 min at 4°C. After two additional wash procedures, substrate solution was added to each well, and plates were further incubated for 30 min at room temperature in the dark, at which point stop solution was added to all wells. Finally, the resulting color was assayed at 450 nm using a microplate absorbance reader (SunriseTM, TECAN, Switzerland).

### Statistical analysis

Statistical analysis of the data was carried out using analysis of variance (ANOVA) for repeated measures followed by Dunnette's post-hoc analysis using GraphPad Prism 4 software (Version 4.03, GraphPad software, Inc., La Jolla, USA).

## Results

### Effect of 4-*O*-methylhonokiol on LPS-induced memory impairment

**Figure 2:**
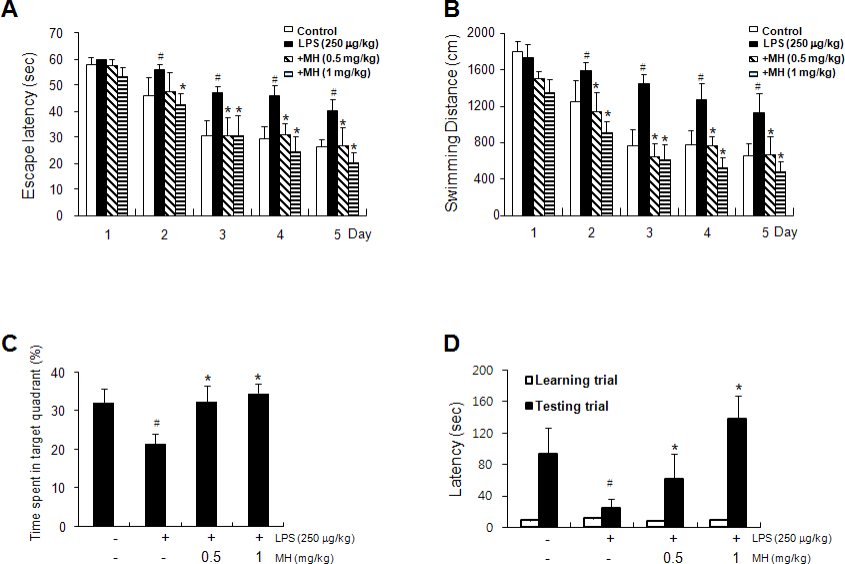
**Effect of 4-*O*-methylhonokiol in LPS-induced memory impairment**. To investigate effect of 4-*O*-methylhonokiol in LPS-induced memory impairment, we performed water maze test (A, B), probe test (C) and step-through type passive avoidance test (D). Memory function was determined by the escape latencies (A, sec) and distance (B, cm) for 5 days, and time spent in target quadrant (C,%) in probe test after administration of LPS. Each value is mean ± S.E. from 10 mice. #, Significantly different from control group (*p *< 0.05). *, Significantly different from LPS-treated group (*p *< 0.05). Control, saline-treated group. LPS, lipopolysaccharide. MH, 4-*O*-methylhonokiol.

To investigate the memory-improving effects by 4-*O*-methylhonokiol on the LPS-induced memory impairment model, mice were continuously administered 4-*O*-methylhonokiol at a dose of 0.5 or 1 mg/kg/day daily for 3 weeks (from day 1 to day 28), and then by i.p. injection with 250 μg/kg/day LPS daily for 1 week (from day 22 to day 28) as shown in Figure 1B. The mice were trained in the Morris water maze test of 15 times training (3 times per day for 5 days). After training, LPS-treated mice slowly arrived at the location of the platform, thus demonstrating memory impairments compared to the control group, but 4-*O*-methylhonokiol significantly ameliorated these memory-impaired effects of LPS-injected mice on escape latencies (in both cm and s) (Figure 2A and 2B). Swimming speed did not differ among the groups (data not shown). The mice exhibited shorter and shorter escape latencies at the end of the training trial, and the average swimming distance and escape latency at the end of training to the platform were about 664.7 ± 133.2 cm and 26.4 ± 2.6 s after 15 times training trial in the control (saline) group. LPS-injected mice exhibited average swimming distance and escape latency to the platform about 1125.2 ± 211.4 cm and 40.2 ± 4.0 s (Figure 2A and 2B). However, the mice that were given 0.5 and 1 mg/kg of 4-*O*-methylhonokiol at day 5 showed a significant and dose-dependent decrease to 669.9 ± 200.6 cm and 26.6 ± 6.9 s, and 473.5 ± 114.2 cm and 20.4 ± 3.7 s, respectively.

One day after the water maze test, we performed a probe trial to measure the maintenance of memory function. During this trial, the average time spent on the target quadrant was decreased in the LPS-injected mice (21.57 ± 2.57%) compared to the control mice (32.13 ± 3.60%), but administration of 4-*O*-methylhonokiol (0.5 or 1 mg/kg/day) in the memory impaired mice significantly increased average time spent to 32.36 ± 4.27% and 34.62 ± 2.40% (Figure 2C). one day after the probe trial, a step-through test was performed. Treatment with LPS (250 μg/kg i.p.) significantly decreased the step-through latency (as determined by passive-avoidance performance in comparison to the control group); however, 4-*O*-methylhonokiol significantly prevented this decreased step-through latency (Figure 1D). The control group exhibited an average step-through latency of 93.6 ± 33.3 s in the illuminated compartment, whereas that of the LPS-treated group decreased to 24.8 ± 11.5 s. 4-*O*-methylhonokiol-treated mice were recovered to 62.1 ± 31.9 s and 138.4 ± 29.7 s from the LPS-induced step-through latency in a dose-dependent manner (Figure 2D).

### 4-*O*-methylhonokiol inhibits LPS-induced iNOS and COX-2 expression

**Figure 3:**
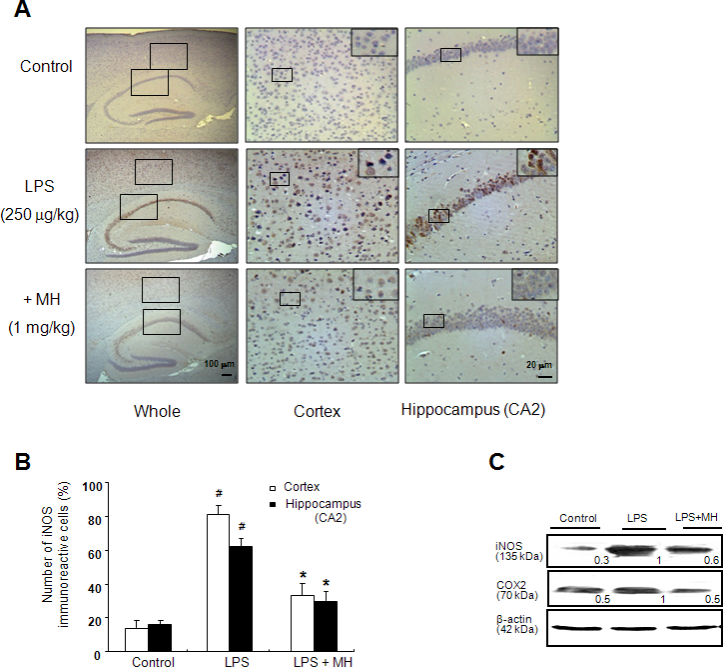
**Inhibitory effects of 4-*O*-methylhonokiol on the LPS-induced expression of inflammatory proteins**. (A) Immunoreactive cells of iNOS antibody were detected in the cortex and hippocampus. 5 μm-thick sections of brains from mice were incubated with anti-iNOS antibodies and the biotinylated secondary antibody. It was then counterstained by hematoxylin. The resulting tissue was viewed with a microscope. (B) The present figure is representative for three different experiments with different animal brains. (C) The expression of iNOS and COX-2 were detected by western blotting using specific antibodies. β-Actin protein was used here as an internal control. The values in the western blot band indicate average density over β-actin from 5 animals. #, Significantly different from control group (*p *< 0.05). *, Significantly different from LPS-treated group (*p *< 0.05). Control, saline-treated group. LPS, lipopolysaccharide. MH, 4-*O*-methylhonokiol.

To investigate the inhibitory effect of 4-*O*-methylhonokiol on memory impairment via inhibition of neuroinflammation, the expression of iNOS was determined by immunohistochemical analysis. Upon LPS treatment, the number of brown-colored (iNOS-labeled) cells in both the cortex and hippocampus of LPS-injected mice was significantly higher than those in control mice, but 4-*O*-methylhonokiol treatment lowered this number (Figure 3A and 3B). Paralleled with the expression level of iNOS detected by immunohistochemical analysis, western blot analysis showed that iNOS expression was significantly increased by LPS injection in mice brain while densitometry data showed that LPS-induced iNOS expression was significantly inhibited by 4-*O*-methylhonokiol (Figure 3C). Since NO can induce COX-2 expression, and COX-2 is also an enzyme that regulates inflammation, we investigated the expression of COX-2 and found that 4-*O*-methylhonokiol also inhibited the LPS-induced COX-2 expression (Figure 3C).

### Effect of 4-*O*-methylhonokiol against the Aβ_1-42 _accumulation in LPS-injected mice brain

**Figure 4:**
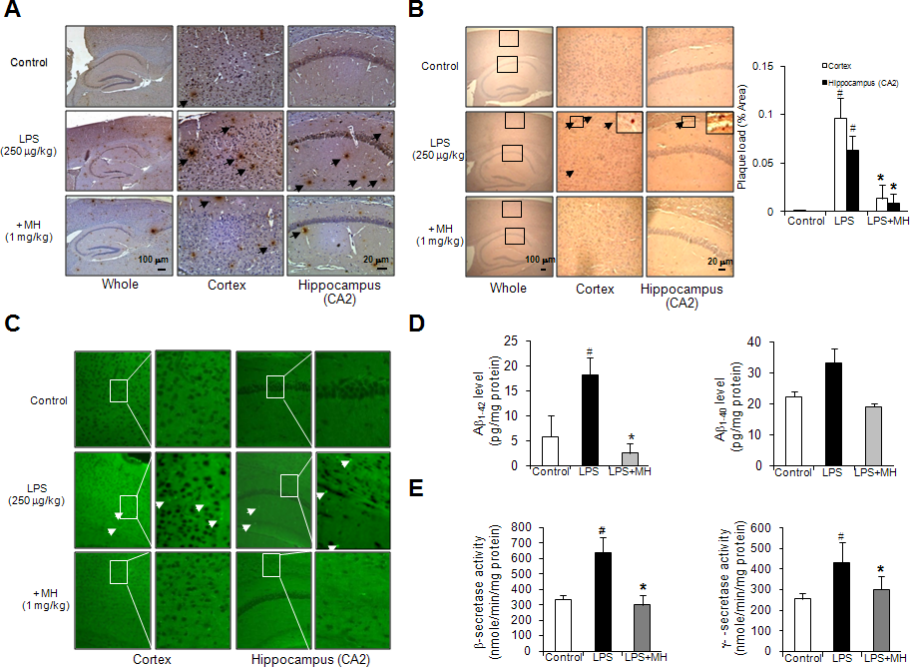
**Effect of 4-*O*-methylhonokiol on Aβ accumulation in the cortex and hippocampus**. (A) Immunoreactive protein of anti-Aβ_1-42 _antibody was investigated in the cortex and hippocampus. 6 μm-thick sections of brains from mice were incubated with anti-Aβ_1-42 _antibody and counterstained with hematoxylin. Arrow indicates Aβ_1-42 _accumulation which is clearly higher in the cerebral cortex and hippocampus of LPS-injected mouse. Amyloid plaque detection via congo red staining (B) and thioflavin S (C) was performed in the cortex and hippocampus. 6 μm-thick sections of brains were incubated with 0.2% congo red solution or thioflavin S solution for 20 min and counterstained with hematoxylin. Arrow indicates amyloid plaque which is clearly higher in the cerebral cortex and hippocampus of LPS-injected mouse. The histograms depict the mean congophilic plaque load ± SEM in mice brain. (D) The levels of Aβ_1-42 _and Aβ_1-40 _were assessed by using a specific Aβ ELISA as described in Methods. (E) The activity of β- and γ-secretase was investigated by using each assay kit as described in Methods. Values measured from each group of mice were calibrated by amount of protein and expressed as mean ± S.E. (n = 5). The values in the western blot band indicate average density over β-actin from three animals. #, Significantly different from control group (*p *< 0.05). *, Significantly different from LPS-treated group (*p *< 0.05). Control, saline-treated group. LPS, lipopolysaccharide. MH, 4-*O*-methylhonokiol.

**Figure 5:**
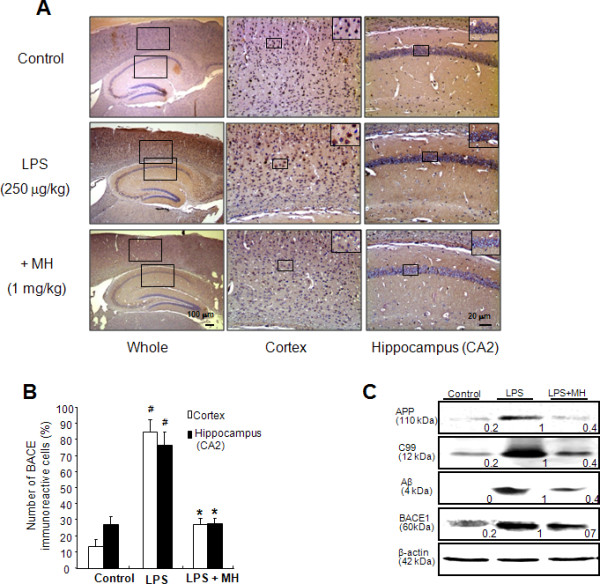
**Effect of 4-*O*-methylhonokiol on expression of amyloidogenic proteins**. (A) Immunoreactive cells of anti-BACE1 antibody were investigated in the cortex and hippocampus. (B) The present figure is representative for three different experiments with different animal brains. (C) The expression of APP, C99 and BACE1 were detected by western blotting using specific antibodies in mice brain. β-Actin protein was used as an internal control. Each blot is representative for three experiments. The values in the western blot band indicate average density over β-actin from 5 animals. #, Significantly different from control group (*p *< 0.05). *, Significantly different from LPS-treated group (*p *< 0.05). Control, saline-treated group. LPS, lipopolysaccharide. MH, 4-*O*-methylhonokiol.

We determined the effect of 4-*O*-methylhonokiol on the levels of Aβ_1-42 _in the cerebral cortex and hippocampus of LPS-injected mice, since the accumulation of Aβ_1-42 _has been implicated in memory dysfunction. We found that, similar to previous data [42], the number of brown colored (Aβ_1-42_-labeled) cells in both the cortex and hippocampus of LPS-injected mice was significantly higher than that in control mice, but 4-*O*-methylhonokiol treatment lowered the increased number of Aβ_1-42_-labeled cells (Figure 4A). In addition, these results could confirm the LPS-induced increase of amyloid plaque using congo red and thioflavin S, and confirm that 4-*O*-methylhonokiol decreases amyloid plaque (Figure 4B and 4C). Paralleled with the reduced Aβ_1-42 _reactive cell number, the level of Aβ_1-42 _(Figure 4D) and the activity of β- and γ-secretases (Figure 4E) were also significantly reduced in 4-*O*-methylhonokiol-treated whole brains of LPS-injected mice. Moreover, the expression of the neuronal BACE1 as well as reactive cell number of BACE1 was significantly reduced by the treatment of 4-*O*-methylhonokiol (Figure 5A and 5B). To confirm these results, we investigated the levels of APP, C99, and BACE1 proteins using western blot analysis. Both the expression level of APP and C99 increased in the brains of LPS-injected mice, and these elevations were reduced by the treatment of 4-*O*-methylhonokiol (Figure 5C).

### Effect of 4-*O*-methylhonokiol on the activation of astrocytes and microglia in LPS-injected mice brain

**Figure 6:**
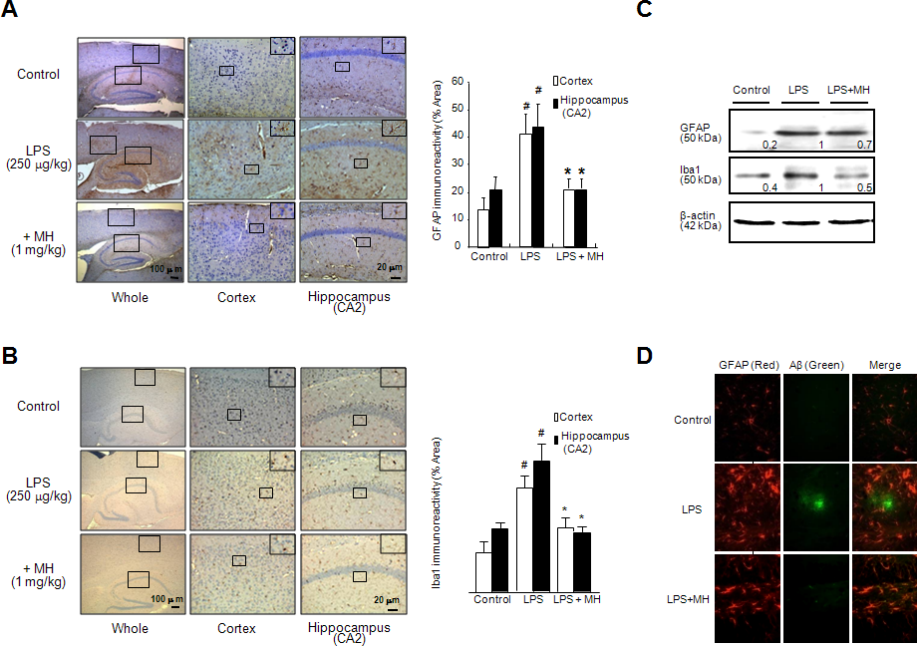
**Effect of 4-*O*-methylhonokiol on the LPS-induced neuroinflammation**. (A) Immunoreactive cells of anti-GFAP antibody were investigated in the cortex and hippocampus. (B) Immunoreactive cells of anti-Iba1 antibody were investigated in the cortex and hippocampus. The present figure is representative for three different experiments with different animal brains. (C) The level of GFAP and Iba1 was detected by western blotting using specific antibodies in mice brain. β-Actin protein was used as an internal control. Each blot is representative for 3 experiments. The values in the western blot band indicate average density over actin from 5 animals. (D) Co-immunoreactivity against anti-GFAP antibody (red label) and anti-Aβ antibody (green label) was investigated. The figure representative of three experiments from different mice brain. #, Significantly different from control group (*p *< 0.05). *, Significantly different from LPS-treated group (*p *< 0.05). Control, saline-treated group. LPS, lipopolysaccharide. MH, 4-*O*-methylhonokiol.

Activation of neuroglia has also been implicated in amyloidogenesis and neuronal cell death during the development of AD [57]. To investigate the protective effect of 4-*O*-methlyhonokiol on activation of astrocytes and microglia, we performed an immunohistochemical analysis of GFAP- and Iba1-reactive cells in the brain. In the LPS-injected mice, GFAP- and Iba1-reactive cell numbers were significantly higher whereas the treatment of 4-*O-*methlylhonokiol reduced the number of GFAP reactive cells in the cortex and hippocampus (Figure 6A and 6B). Paralleled with the immunohistochemical results, western blot analysis also showed that GFAP and Iba1 levels were increased in the brains of LPS-injected mice, and these levels were then reduced by the treatment of 4-*O*-methylhonokiol (Figure 6C). To demonstrate more clearly that the activation of astrocytes could cause Aβ generation, co-immunoreactive cells against GFAP and Aβ_1-42 _were identified by means of double immunofluorescence method (Figure 6D). The co-reactive cell number for both markers was significantly increased by LPS, but was lowered by 4-*O-*methylhonokiol treatment.

**Figure 7:**
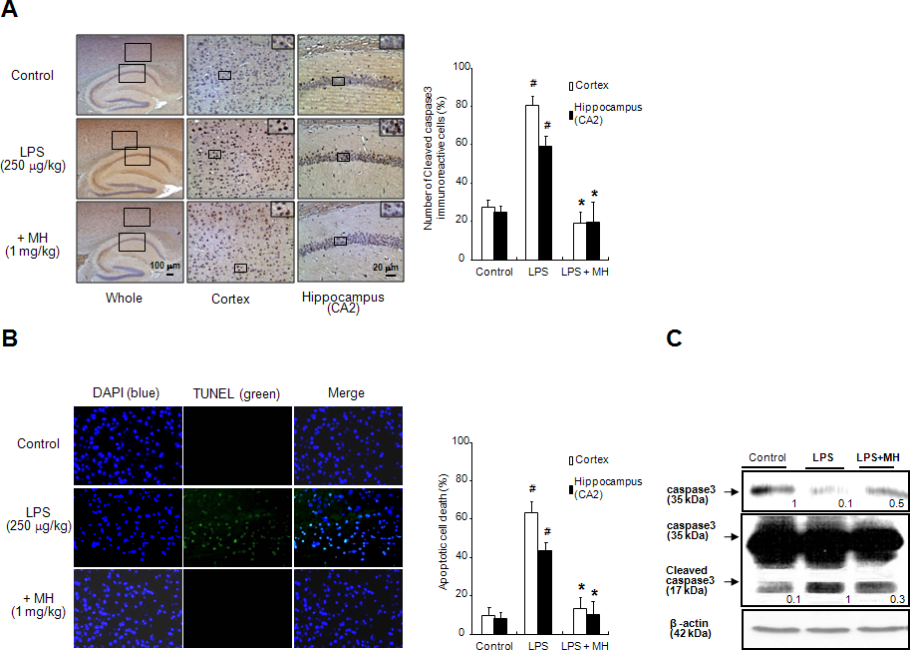
**Effect of 4-*O*-methylhonokiol on the LPS-induced neuronal cellular damage**. (A) Immunoreactive cells of anti-cleaved caspase-3 antibody were investigated in the cortex and hippocampus. The present figure is representative for 3 different experiments with different animal brains. (B) Apoptotic cell death was determined by DAPI staining and TUNEL assay as described in Methods. Each panel represents 6 animals. (C) The levels of cleaved caspase-3 and caspase-3 were detected by western blotting using specific antibodies in mice brain. β-Actin protein was used as an internal control. The values in the western blot band indicate average density over β-actin from 5 animals. #, Significantly different from control group (*p *< 0.05). *, Significantly different from LPS-treated group (*p *< 0.05). Control, saline-treated group. LPS, lipopolysaccharide. MH, 4-*O*-methylhonokiol.

Next, to investigate both the consequence of neuroglia activation and amyloidogenesis by LPS and the protective effect of 4-*O*-methylhonokiol, cell death was investigated by determining the expression levels of the pro-apoptotic protein, cleavage caspase-3. LPS-injection induced higher expressions of cleavage caspase-3, but treatment with 4-*O*-methylhonokiol decreased this expression as determined by immunohistochemical (Figure 7A) and western blot analysis (Figure 7C). In addition, to confirm LPS-induced neuronal cell death, we performed TUNEL assay. Paralleled with results of cleavage caspase-3, LPS-injection increased number of apoptotic neuronal cells, and treatment with 4-*O*-methylhonokiol decreased number of apoptotic cells.

### Effect of 4-*O*-methylhonokiol on LPS-induced release of NO, PGE_2_, ROS, TNF-α and IL-1β in cultured astrocytes

**Figure 8:**
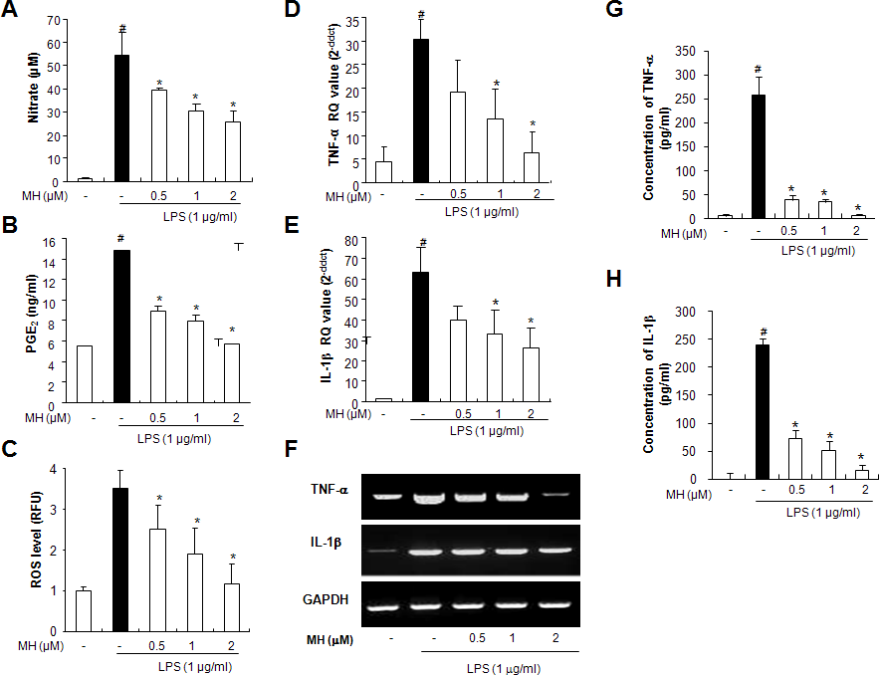
**Effect of 4-*O*-methylhonokiol on LPS-induced ROS, NO, PGE_2_, TNF-α and IL-1β generation in cultured astrocytes**. Astrocytes were treated with LPS (1 μM) and 4-*O*-methylhonokiol (0.5-2 μM). (A) NO level was determined in the supernatant of astrocytes by Griess reaction as described in Methods. (B) PGE_2 _level was determined in the supernatant of astrocytes by PGE_2 _EIA kit. (C) Intracellular ROS levels were determined by measuring DCF fluorescence. (D), (E) and (F) mRNA levels of TNF-α and IL-1β were determined by real time PCR as described in Methods. (G) and (H) protein levels of TNF-α and IL-1β were determined by specific ELISA kits as described in Methods. The data indicated in the each band are means ± S.D. from 5 mice brains. Values represent means ± S.D. of three independent experiments performed in triplicate. #, Significantly different from control group (*p *< 0.05). *, Significantly different from LPS-treated group (*p *< 0.05). Control, saline-treated group. LPS, lipopolysaccharide. MH, 4-*O*-methylhonokiol.

To further investigate the anti-neuroinflammatory and anti-amyloidogenesis effects of 4-*O*-methylhonokiol in cultured astrocytes, which are responsible for neuroinflammation and amyloidogenesis in the brain, we examined the inhibitory effect of 4-*O*-methylhonokiol on the LPS (1 μg/ml)-induced NO and PGE_2 _production as well as expression of iNOS and COX-2 in cultured astrocytes. After co-treatment with LPS and 4-*O*-methylhonokiol (0.5, 1 and 2 μM) for 24 h, LPS-induced nitrate concentrations in the medium were decreased remarkably in a concentration-dependent manner (Figure 8A). We further investigated the effects of 4-*O*-methylhonokiol on the LPS-induced synthesis of PGE_2_, the major product of COX-2 enzymatic activity. While LPS induced a marked synthesis of PGE_2_, 4-*O*-methylhonokiol blocked LPS-induced PGE_2 _synthesis (Figure 8B). To study the protective effect of 4-*O*-methylhonokiol against the LPS-induced activation of astrocytes, we investigated the release of ROS, TNF-α and IL-1β was determined as indicators of astrocytes activation as well as inflammatory responses. We found that treatment of 4-*O*-methylhonokiol reduced LPS-induced ROS generation (Figure 8C). LPS-induced TNF-α and IL-1β release in cultured astrocytes was also reduced by 4-*O*-methylhonokiol in a concentration-dependent manner (Figure 8D, E, F, G and 8H).

Effect of 4-*O*-methylhonokiol on LPS-induced NF-κB transcriptional and DNA-binding activity as well as iNOS and COX-2 expression in cultured astrocytes

**Figure 9:**
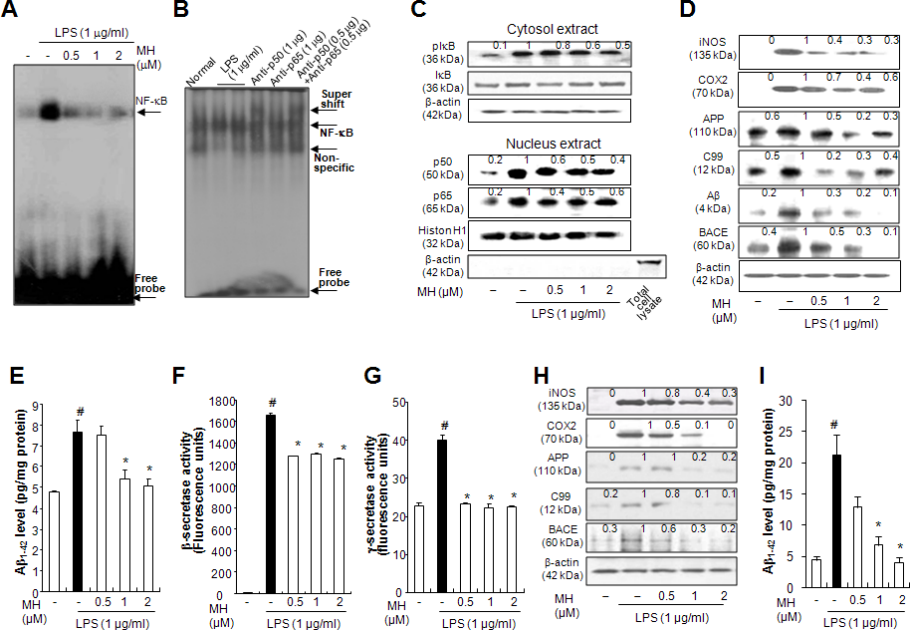
**Effect of 4-*O*-methylhonokiol on LPS-induced iNOS, COX-2, APP, C99, Aβ, BACE generation, NF-κB activity, β- and γ-secretase activity in cultured astrocytes and in microglial BV-2 cells**. Astrocytes and microglial BV-2 cells were treated with LPS (1 μM) and 4-*O*-methylhonokiol (0.5-2 μM). (A) NF-κB activity in astrocytes was determined by EMSA as described in Methods. (B) For supershift assays, nuclear extracts from cultured astrocytes treated with LPS (1 μg/ml) were incubated for 1 h before EMSA with specific antibodies against the p50 and p65 NF-κB isoforms. (C) Phosphorylation of IκB, and p50 and p65 translocation in the astrocytes. (D) Immunoblots of lysates from astrocytes were probed with iNOS, COX-2, APP, C99, Aβ and BACE antibodies, respectively. (E) The amounts of Aβ_1-42 _were assessed by using a specific Aβ_1-42 _ELISA kit as described in the Methods. (F) and (G) β- and γ-secretase activity in the astrocytes were determined as described in Methods. (H) Immunoblots of lysates from microglial BV-2 cells were probed with iNOS, COX-2, APP, C99 and BACE antibodies, respectively. (I) The amounts of Aβ_1-42 _were assessed by using a specific Aβ_1-42 _ELISA kit as described in the Methods. Values represent means ± S.D. of three independent experiments performed in triplicate. The values in the western blot band indicate average density over β-actin from three animals. #, Significantly different from control group (*p *< 0.05). *, Significantly different from LPS-treated group (*p *< 0.05). Control, saline-treated group. LPS, lipopolysaccharide. MH, 4-*O*-methylhonokiol.

It has been demonstrated that LPS activates transcription factor NF-κB leading to the increased expression of many immediate-early genes, including the amyloidogenesis-related enzymes, such as BACE1. Thus, our next step was to investigate NF-κB DNA-binding activity. LPS-induced NF-κB DNA binding activity in cultured astrocytes was decreased by 4-*O*-methylhonokiol in a concentration-dependent manner (Figure 9A). This DNA-binding activity of NF-κB was confirmed by super shift assays. In the presence of a p50 antibody, the DNA-binding activities of NF-κB showed a super shift. However, in the presence of a p65 antibody, the DNA-binding activity of NF-κB was decreased without a super shift, suggesting that p50 might be a target of 4-*O*-methylhonokiol, interfering with the DNA-binding activity of NF-κB (Figure 9B). To clarify the action mechanism of 4-*O*-methylhonokiol on NF-κB activity, the nuclear translocation of p50 and p65 was examined. In the presence of 4-*O*-methylhonokiol, LPS-induced nuclear translocation of p50 and p65 in astrocytes was inhibited in a concentration-dependent manner (Figure 9C). Moreover, 4-*O*-methylhonokiol inhibited LPS-induced degradation of IκBα via inhibition of IκBα phosphorylation (Figure 9C). To examine the consequence of the inhibitory effects of 4-*O*-methylhonokiol on NF-κB activity, expression of the NF-κB-derived inflammatory genes, iNOS and COX-2, was investigated. As shown in Figure 9D, the cells expressed extremely low levels of iNOS in the unstimulated condition. However, iNOS expression was markedly increased in response to LPS after 24 h. Treatment with 4-*O*-methylhonokiol (0.5, 1 and 2 μM) concentration-dependently decreased LPS-induced expression of iNOS in cultured astrocytes. These results indicate that 4-*O*-methylhonokiol may inhibit the LPS-induced activation of NF-κB via inhibition of IκBα degradation, as well as via the translocation of p50 and p65 into the nucleus, and this effect may result in the inhibition of LPS-induced expression of iNOS and COX-2.

### Effect of 4-*O*-methylhonokiol on LPS-induced amyloidogenesis in cultured astrocytes and microglial BV-2 cells

It is known that the activation of microglia and astrocytes are major sources of neuro-inflammation. Increasing evidence indicates that inflammatory stimuli concomitantly increase amyloidogenesis in astrocytes. Thus, we investigated whether 4-*O*-methylhonokiol prevented LPS-induced amyloidogenesis. Low levels of APP, BACE and C99 protein were found in the unstimulated control condition, whereas the expression of BACE, APP and C99 proteins increased in response to LPS (1 μg/ml) after 24 h. Treatment with 4-*O*-methylhonokiol (0.5, 1 and 2 μM) caused a concentration-dependent inhibition in LPS-induced BACE, C99 and APP expression in astrocytes and in microglial BV-2 cells (Figure 9D and 9H). We also determined the effect of 4-*O*-methylhonokiol on the levels of Aβ_1-42 _in the LPS-stimulated astrocytes and microglial BV-2 cells, and found a significantly higher level of Aβ_1-42 _in the LPS group compared to levels in control cells. 4-*O*-methylhonokiol treatment, however, lowered this increased level of Aβ_1-42 _(Figure 9E and 9I). Paralleled with the reduced level of Aβ_1-42_, activity of β- and γ-secretase were also significantly reduced by 4-*O*-methylhonokiol in LPS-stimulated astrocytes (Figure 9F and 9G).

## Discussion

The most important finding of this study is that 4-*O*-methylhonokiol, a lignan compound isolated from *Magnolia officinalis*, suppressed amyloidogenesis via its anti-neuroinflammatory properties in LPS-induced *in vivo *and *in vitro *model, and ameliorated memory impairment. Accumulating epidemiological evidence has suggested that neuroinflammation may contribute to the occurrence and progression of AD [8,9,58-60], as brains of AD patients appear to display hallmarks of neuroinflammation, including marked astrogliosis, elevated release of proinflammatory mediators and cytokines, and microglial activation [61,62]. Recently, several researchers were reported that systemic administration of LPS induces release of proinflammatory mediators and cytokines such as TNF-α, IL-1β, iNOS, COX-2, cytosolic group IV phospholipase A2, 5*-*lipoxygenase and toll-like receptor-4 [49,50,63], indicating that systemic inflammation induces neuroinflammation. Moreover, systemic administration of LPS has been reported to result in increased APP processing and intracellular accumulation of Aβ as well as memory deficiency with concomitant increased neuroinflammation [27,28,64,65]. In particular, Jaeger et al. [51] reported that repeated systemic injections (3 times) of LPS increased Aβ accumulation in the brain through increased influx of blood Aβ contributed to alteration of LRP-1 in mice brain although it is different with our mechanism of amyloid deposition, and it supports our LPS-induced AD model. Administration of non-steroidal anti-inflammatory drugs (NSAIDs) could reduce the risk and delay the onset of AD [25,60,66]. Thus, anti-inflammatory agents could decrease amyloidogenesis and memory deficiency via the prevention of neuroinflammation.

The results of our previous studies [27,28] and the present one showed that inflammatory and amyloidogenic genes were concomitantly increased by treatment with LPS. However, 4-*O*-methylhonokiol has resulted in the inhibition of the expression of the NF-κB and NF-κB-mediated expression of inflammatory proteins; COX-2 and iNOS as well as ROS, NO, PGE_2_, TNF-α and IL-1β levels in the brain and in cultured astrocytes. These inhibitory effects are in agreement with the inhibitory effects on the expression of proteins involved in amyloidogenesis, such as BACE1 and C99, a product of BACE1. 4-*O*-methylhonokiol also inhibited the proteolytic cleavage of APP via the inhibition of β- or γ-secretase activity, resulting in the reduction of Aβ generation. Activation of astrocytes has been known to increase β-secretase activity, thereby increase Aβ generation [67-69]. In fact, we found that GFAP (activation of astrocytes) was co-localized with Aβ, and this co-stimulation was increased by LPS, but was prevented by 4-*O*-methylhonokiol. BACE1 expression was detectable as early as the morphological features of reactive astrocytes. The astrocytic expression of BACE1 after induction of chronic gliosis was not only limited to experimental animals but also included astrocytes in close proximity to β-amyloid plaques in the brains of AD patients [70]. Thus, the present data indicated that the anti-inflammatory properties of 4-*O*-methylhononkiol could be associated with anti-amyloidogenesis.

It is not clear how 4-*O*-methylhonokiol concomitantly reduces neuroinflammation and amyloidogenesis. However, it is noteworthy that ROS and NO have been implicated in the activation of BACE1 expression as well as in the activity of β- and γ-secretases [71,72]. In a recent study, the role of ROS release by the mitochondrial electron chain in response to hypoxia was determined to foster amyloidogenic APP processing via the up-regulation of β-secretase activity [72]. Additionally, we previously found that compounds having antioxidant properties such as EGCG and L-theanine showed anti-neuroinflammatory responses and anti-amyloidogenesis activity via anti-oxidant mechanisms [27,73]. Anti-oxidants such as superoxide dismutase [74], α-lipoic acid [75], S-nitrosoglutathione [76], curcumin and docosahexaenoic acid [77], also prevented cognitive deficits, oxidative damage and amyloidogenesis in AD models. Inflammatory reactions have also been known to directly regulate amyloidogenesis via the modification of β-secretase activity [78]. LPS-treated mice exhibited increases in both the levels of inflammatory products and amyloid formation as well as in secretases activity in the neurons of the mouse brain [64]. We also found that 4-*O*-methylhonokiol prevented the activation of astrocytes in culture or in the brain of AD-model mice such as Aβ-injected or PS2 mutant transgenic mice [43,44,48]. Moreover, several anti-inflammatory agents have been reported to have anti-amyloidogenic effects. The administration of minocycline in 8-month-old 3xTg-AD mice was also shown to prevent cognitive deficits and to decrease insoluble Aβ and soluble fibrils via the reduction of inflammatory agents such as GFAP, TNF-α and IL-6 [79]. Furthermore, ibuprofen was reported to reduce Aβ_1-42 _production via the inhibition of γ-secretase [80] and the reduction of neuroinflammation via COX inhibition [81]. Ibuprofen also decreased β-secretase activity via inhibition of peroxisome proliferator-activated receptor gamma [82], cytokines, α-1-antichymotrypsin [83] and the Rho cascade [84,85]. Thus, anti-oxidative and anti-inflammatory properties of 4-*O*-methylhonokiol could be significant for anti-amyloidogenesis.

The generation of Aβ requires the proteolytic cleavage of APP by an aspartyl protease named BACE1 [86]. A number of transcriptional factors as well as post-transcriptional modifications and intracellular signaling molecule activation, regulate BACE1 expression in the brain [87]. The expression of endogenous BACE1 proteins in differentiated PC12 cells was decreased by the pharmacological inhibition of NF-κB activation via (R)-flurbiprofen and by treatment with decoy oligonucleotides that were specific for the BACE1 protein promoter NF-κB site [87,88]. NF-κB has also been well documented in decreasing transcription factors regulating β-secretase in activated astrocytes [89,90]. The promoters of APP [91], presenilin and BACE1 [92] contain NF-κB sites, which derive transcription. Some NSAIDs such as flurbiprofen and indomethacin, which target NF-κB, have been shown to be effective at decreasing amyloid load *in vitro *and also in APP transgenic mice [93-95]. In addition, numerous factors were reported to inhibit amyloidogenesis via suppression of NF-κB such as sorafenib [96], L-theanine [73], and tripchlorolide [97]. We previously found that activation of NF-κB contributes to the increase in β-secretase in neuronal cells expressing mutant PS2 [44], and also demonstrated that EGCG, as a well known anti-oxidant and anti-inflammatory agent, inhibits β- and γ-secretases activity via inhibition of NF-κB pathways in PS2 mice [42]. It was also illustrated that 4-*O*-methylhonokiol had an anti-inflammatory effect in LPS-induced RAW 264.7 cells via inhibition of NF-κB pathway activation [35]. In this study, 4-*O*-methylhonokiol inhibited dose-dependent LPS-induced activation of the NF-κB pathway in astrocytes, and this inhibition of NF-κB pathway resulted in a dose-dependent decrease in the nuclear translocation of the p50 and p65 subunits, and it also decreased phosphorylation of IκB in astrocytes. Thus, inactivation of NF-κB signaling pathways in the control of β- and γ-secretase by 4-*O*-methylhonokiol could be critical in the reduction of these secretases, and thus the inhibition of Aβ generation.

We previously performed the pharmacokinetic study in ICR mice. We treated by direct compulsory oral administration (10 mg/kg). We found that oral treatment of 4-*O*-methylhonokiol rapidly disappears from the blood and is distributed into brain rapidly (less than 1 hr after treatment) (unpublished data). The blood concentration of 4-*O*-methylhonokiol gets platue after 2 hr treatment which is similar level with the concentration by intravenous injection. The effective dose of blood may be about 20 ng/ml. The tissue concentration pharmacokinetic profile showed that the 4-*O*-methylhonokiol could be accumulated into brain, and about 50-100 ng/ml may be effective dose in the brain. Moreover, oral administration of 4-*O*-methylhonokiol of as much as 80 mg/kg for 4 weeks did not cause weight loss or other toxicities in a repeated-dose toxicity study (unpublished data). 4-*O*-methylhonokiol was also evaluated as noncarcinogenic in rodents (data not shown). These data suggest that it could be safe and effective in a clinical application.

## Conclusion

Our data show that 4-*O-*methylhonokiol has ameliorated LPS-induced memory deficiencies via the inhibition of Aβ_1-42 _generation, by inactivating β- and γ-secretases and astrocytes via the inactivation of NF-κB pathways. This study therefore suggests that 4-*O-*methylhonokiol may be a useful agent for preventing the development or progression of AD.

## Abbreviations

Aβ: Amyloid beta-peptide; AD: Alzheimer's disease; APP: Amyloid precursor protein; BACE1: β-secretase-1; DAPI: 4'-6-diamidino-2-phenylindole; DCF-DA: 2',7'-Dichlorofluorescein diacetate; DMEM: Dulbecco's modified Eagle's medium; EGCG: (-)-Epigallocatechin 3-gallate; ELISA: Enzyme-linked immunosorbent assay; EMSA: Electrophoretic mobility shift assay; FBS: Fetal bovine serum; GAPDH: Glyceraldehyde-3-phosphate dehydrogenase; GFAP: Glial fibrillary acidic protein; Iba1: Ionized calcium binding adaptor molecule 1; ICR: Imprinting control region; IL: Interleukin; i.p.: intraperitoneal; iNOS: Inducible nitric oxide synthase; LPS: Lipopolysaccharide; NF: Nuclear factor; NMR: Nuclear magnetic resonance; NO: Nitric oxide; NSAIDs: Nonsteroidal anti-inflammatory drugs; PBS: Phosphate-buffered saline; PCR: Polymerase chain reaction; PGE_2_: Prostaglandin E2; ROS: Reactive oxygen species; SD: Standard deviation; TNF: Tumor necrosis factor; TUNEL: Terminal deoxynucleotidyl transferase-mediated dUTP-biotin nick end-labeling.

## Competing interests

The authors declare that they have no competing interests.

## Authors' contributions

Hong JT designed the study. Lee YJ prepared the manuscript and performed experiments. Choi DY and Choi IS performed experiments. Kim KH and Kim YH isolated and characterized 4-*O*-methylhonokiol. Kim HM, Lee K, Cho WG, Jung JK, Han SB, Han JY, Nam SY, Yun YW and Oh KW discussed the study. All authors have read and approved the final version of this manuscript.
